# Supplementary material for: Coding and regulatory variants are associated with serum protein levels and disease
Source: Nat Commun. 2022 Jan 25;13:481. doi: 10.1038/s41467-022-28081-6 (PMC8789809; doi:10.1038/s41467-022-28081-6)
Supplement: Supplementary file 1 — Supplementary Information [file 41467_2022_28081_MOESM1_ESM.pdf]

Supplementary Information

Coding and regulatory variants are associated with serum protein levels and disease

Valur Emilsson[1,2,*], Valborg Gudmundsdottir[1,*], Alexander Gudjonsson[1,*], Thorarinn Jonmundsson[2], Brynjolfur G. Jonsson[1], Mohd A Karim[3,4], Marjan Ilkov[1], James R. Staley[5], Elias F. Gudmundsson[1], Lenore J. Launer[6], Jan H. Lindeman[7], Nicholas M. Morton[8], Thor Aspelund[1], John R. Lamb[9], Lori L. Jennings[10] and Vilmundur Gudnason[1,2]

[1]Icelandic Heart Association, Holtasmari 1, IS-201 Kopavogur, Iceland.
[2]Faculty of Medicine, University of Iceland, 101 Reykjavik, Iceland
[3]Wellcome Trust Sanger Institute, Welcome Genome Campus, Hinxton, Cambridgeshire CB10 1SA, UK.
[4]Open Targets, Wellcome Genome Campus, Hinxton, Cambridgeshire CB10 1SD, UK
[5]BHF Cardiovascular Epidemiology Unit, Department of Public Health and Primary Care, University of Cambridge, Cambridge, UK
[6]Laboratory of Epidemiology and Population Sciences, Intramural Research Program, National Institute on Aging, Bethesda, MD 20892-9205, USA.
[7]Department of Surgery Leiden University Medical Center, Leiden. Netherlands
[8]Centre for Cardiovascular Sciences, Queen's Medical Research Institute, University of Edinburgh, Edinburgh EH16 4TJ, UK
[9]GNF Novartis, 10675 John Jay Hopkins Drive, San Diego, CA 92121, USA.
[10]Novartis Institutes for Biomedical Research, 22 Windsor Street, Cambridge, MA 02139, USA.

[*]These authors contributed equally.

Corresponding authors. Emails: valur@hjarta.is and v.gudnason@hjarta.is

Supplementary Note 1: Estimates of novelty for pQTLs reported in the current study

Novel SNP-protein associations: In general, when estimating novelty of SNP-protein associations, the following factors are considered: 1. A novel independent SNP (pQTL) affecting a novel protein; 2. A novel independent pQTL affecting a protein previously associated with different pQTL(s); 3. A previously known pQTL affecting a novel protein. In comparison to all 19 external studies found in the public domain (listed in Supplementary Data 6), the current study significantly increases the number of genetic signals underlying serum proteins. More to the point, using conditionally independent study-wide signficiant associations (Supplementary Data 2) and LD thresold of r^2<0.5 for novel associations, the current study reveals 76.8% novel SNP-protein associations compared to Emilsson et al.[1], 75.5% compared to Sun et al.[2], and with 59.3% of the 4001 SNP-protein associations being novel in comparison to all published pQTL studies (left panel in Supplementary Fig. 6A). These comparisons are also shown using LD thresold of r^2<0.9 for novel associations. Here, the current study finds 81.7% novel SNP-protein associations compared to Emilsson et al.[1], 76.2% compared to Sun et al.[2], and 62.0% novel SNP-protein associations compared to all published pQTL studies (Supplementary Fig. 6A, right panel).

Similarly, when we compared all conditionally independent study-wide significant SNP-protein associations in the new GWAS paper by Gudjonsson et al.[3] with the current exome-array study, using LD of r^2<0.5 for novel associations, we find that 49.5% (2053 of 4147 conditionally independent SNP-protein associations in the GWAS) were GWAS-specific, while 48.4% (1937 of 4001 conditionally independent SNP-protein associations in the exome-array study) were exome-array-specific (left panel in Supplementary Fig. 6B). Using LD of r^2<0.9 for the

comparison, 59.8% were GWAS specific while 58.6% were exome-array specific (right panel in Supplementary Fig. 6B).

Finally, we obtain 6362 SNP-to-protein associations by combining all unique and common SNP-protein signatures from both companion studies. Here, at LD of r^2<0.5 for novel SNP-protein associations, 77.9% were novel compared to Emilsson et al.[1] and 74.6% compared to Sun et al.[2], while at LD of r^2<0.9, 82.3% were novel compared to Emilsson et al.[1] and 77.4% compared to Sun et al.[2]. When compared to external data sets with LD of r^2<0.5 or r^2<0.9, we find that 60.0% and 64.8%, respectively, of the conditionally independent SNP-protein associations presented by our two companion papers are novel (Supplementary Fig. 6C).

<u>Novel locus-protein associations:</u> First, we examined which aptamers were study-wide significant in each of the two companion papers, determining how many are explicitly found in each paper and how many are found in both. We then defined neighboring lead SNP signals to be from the same locus if the distance between the signals was less than 300kb, which is consistent with the window used in our GWAS paper[3] and our previous publication in Science[1]. More specifically, for each study we combined neighboring conditionally independent lead SNP signals into a unified locus until no other SNP signal was within 300kb of the locus, at which point we define a new locus and proceed in the same way. The output of this procedure were two sets of genomic ranges, one for each paper, which we then analyzed to see how many loci overlapped and how many were unique between the two studies. When comparing the papers to previously published proteomic studies, we combined the previous studies into one dataset, performed the same operation on this larger dataset and compared genomic ranges thus obtained to the previously mentioned ranges.

When a locus in one study overlaps with a locus in the other, we consider it shared (independent of if they associate with the same proteins or not). This analysis reveals that the GWAS study by Gudjonsson et al.[3] does not cover 321 of the 881 loci identified in the present exome-array paper (Supplementary Data 7). When the exome array data is compared to the results of the previous 19 pQTL studies (listed in Supplementary Data 6), the current study finds 292 novel loci (Supplementary Data 7). Finally, when the current exome array study and the study by Gudjonsson et al.[3] are combined and compared to all previously published pQTL studies, the two studies yield 404 novel loci (Supplementary Data 7). Next, we looked at locus-protein associations and considered them shared if the locus overlapped with a locus in the other study that was furthermore associated with the same protein. In this study, 762 of 3103 locus-protein associations are unique to the exome-array study when compared to the GWAS paper by Gudjonsson et al.[3]. When the exome array results are compared to all previous pQTL publications (Supplementary Data 6), 1473 locus-protein associations were found to be novel (Supplementary Data 7). Similarly, when compared to previous pQTL publications, the present study and the study by Gudjonsson et al.[3] combined revealed 1950 novel locus-protein associations (Supplementary Data 7). In conclusion, the exome array yields many novel findings at both the locus-protein and SNP-protein levels.

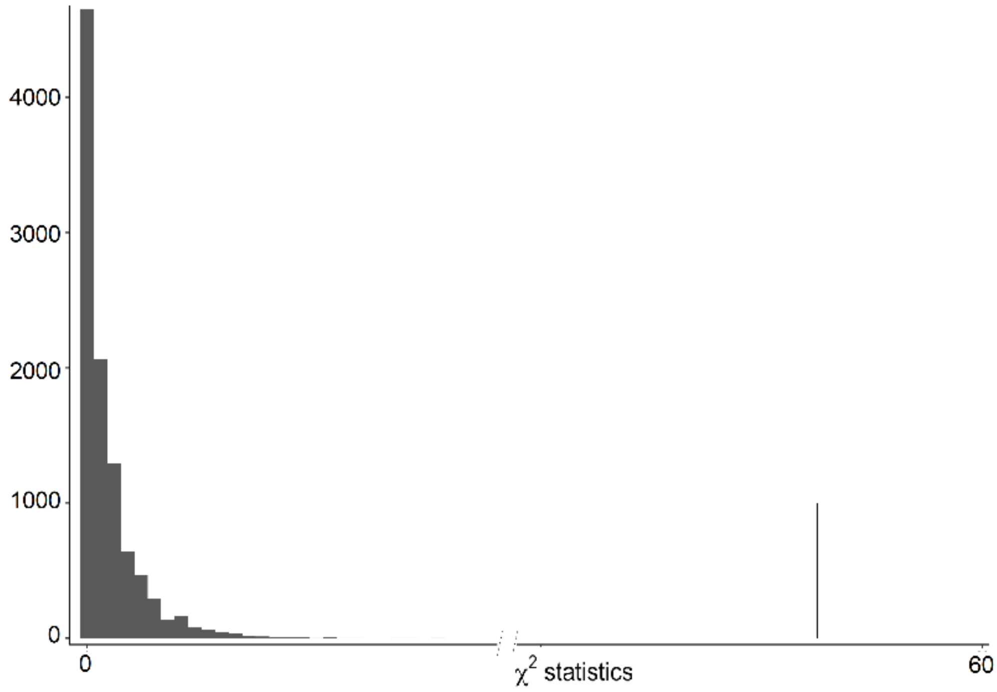

Supplementary Fig. 1. Determine whether the percentage of secreted proteins among pQTLs is equal to the percentage of secreted proteins among non-pQTLs. Empirical distribution of the test statistic as a histogram and the observed statistics calculated from our data as a vertical line. We included information on 4137 proteins, 2021 of which had a pQTL that could be classified as secreted or non-secreted. 10,000 permutations were performed to obtain the empirical distribution of the χ^2 test of equality of proportions of pQTLs among secreted versus non-secreted proteins. Here, the test statistics calculated from our data to the quantiles of this distribution to obtain $P(Data|H_0)$ (see Methods) were compared. Of 10,000 permutations none gave a value greater than the observed statistic leading us to P-value = $P(Data|H_0) < 0.0001$.

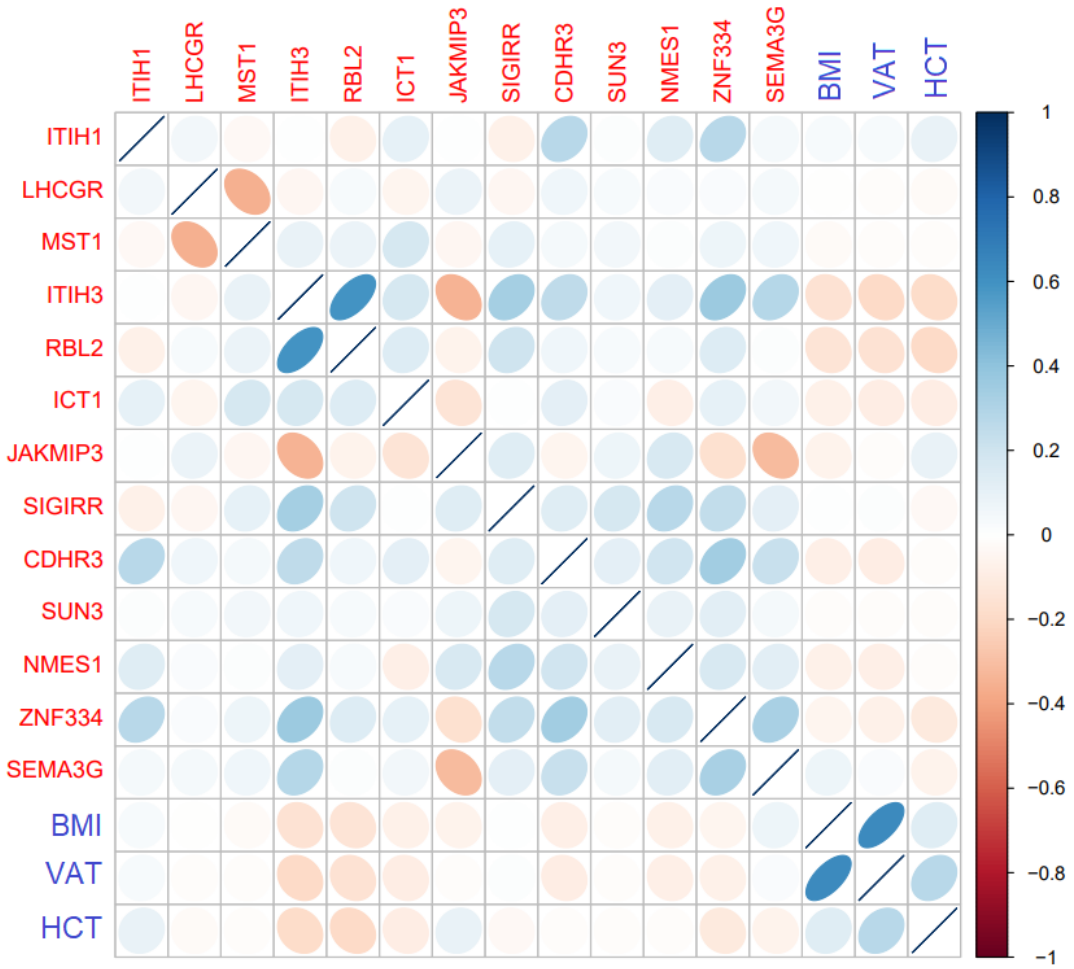

Supplementary Fig. 2. A correlation matrix showing the Spearman rank correlation (Spearman rho metric, the color bar) between all proteins as well as some quantitative traits including body mass index (BMI, kg/m^2), visceral adipose tissue (VAT, measured *via* computed tomography) and hematocrit (HCT), that were associated with rs2251219.

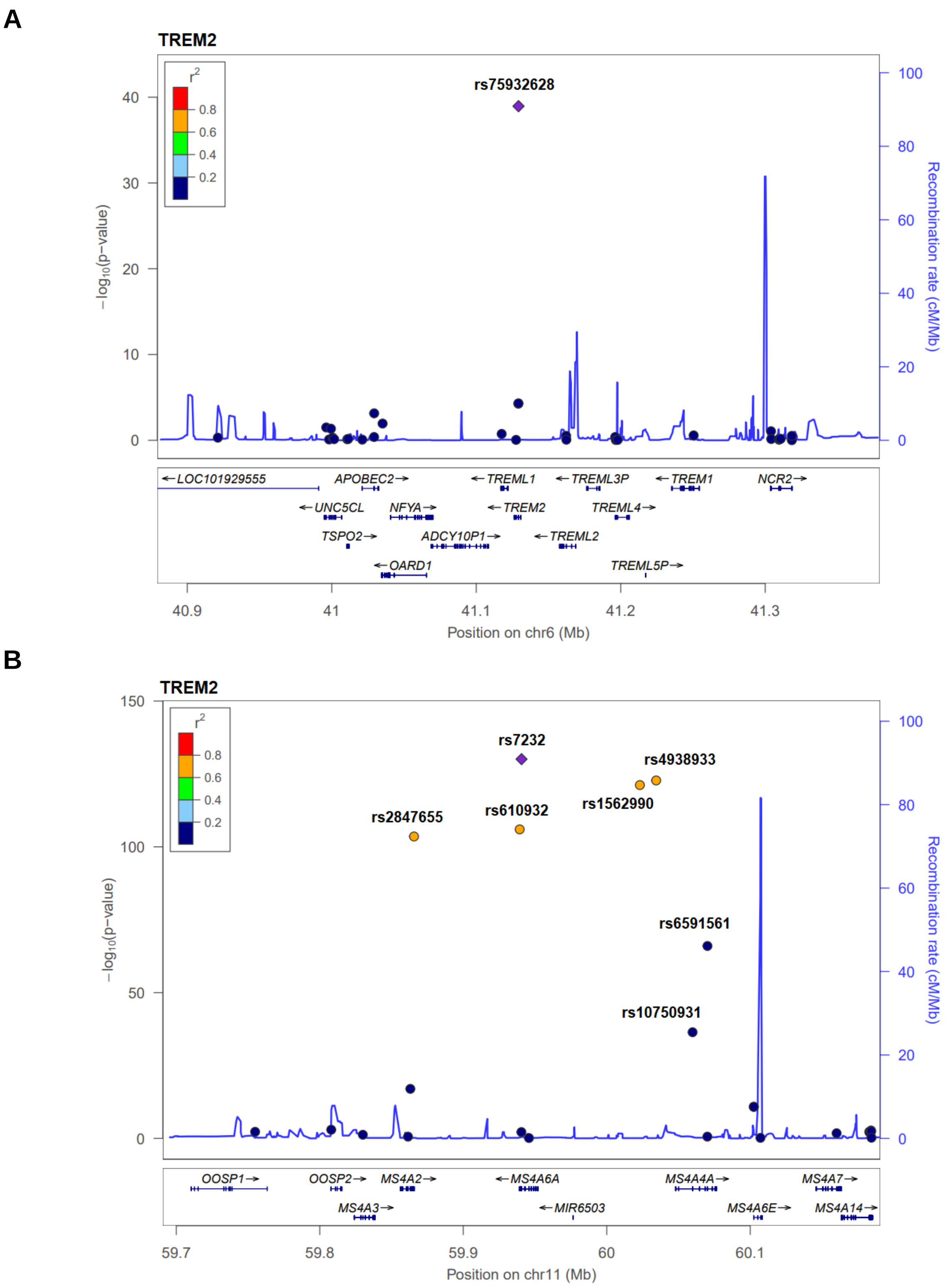

Supplementary Fig. 3. TREM2 regional plots (LocusZoom) based on exome array variants at (**A**) chromosome 6 and (**B**) chromosome 11, using LD data from the AGES-RS cohort. Each plot highlights study-wide significant pQTLs ($P < 1.92\times10^{-10}$, two-sided) pQTLs with

chromosomal location (the megabase position is based on the human genome coordinates version GRCh37/hg19) at the x-axis and -log(P-value) at the y-axis. Datapoints (SNPs) are colored based on their correlation (r^2) with the top SNP, which has the smallest P-value in the region. The fine-scale recombination rates are shown in light blue with genes highlighted below.

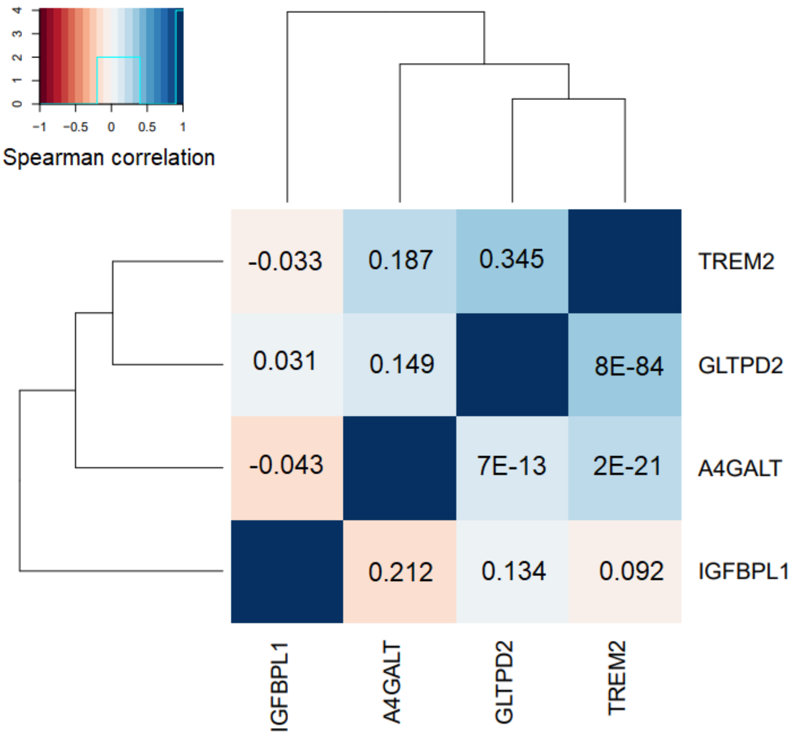

Supplementary Fig. 4. The graph shows the Spearman´s rank correlation between the four serum proteins affected by the two LOAD risk variants, rs75932628 and rs610932. The correlation matrix's upper triangle depicts the rho values, while the lower triangle highlights the P-values (two-sided) of protein-to-protein correlations.

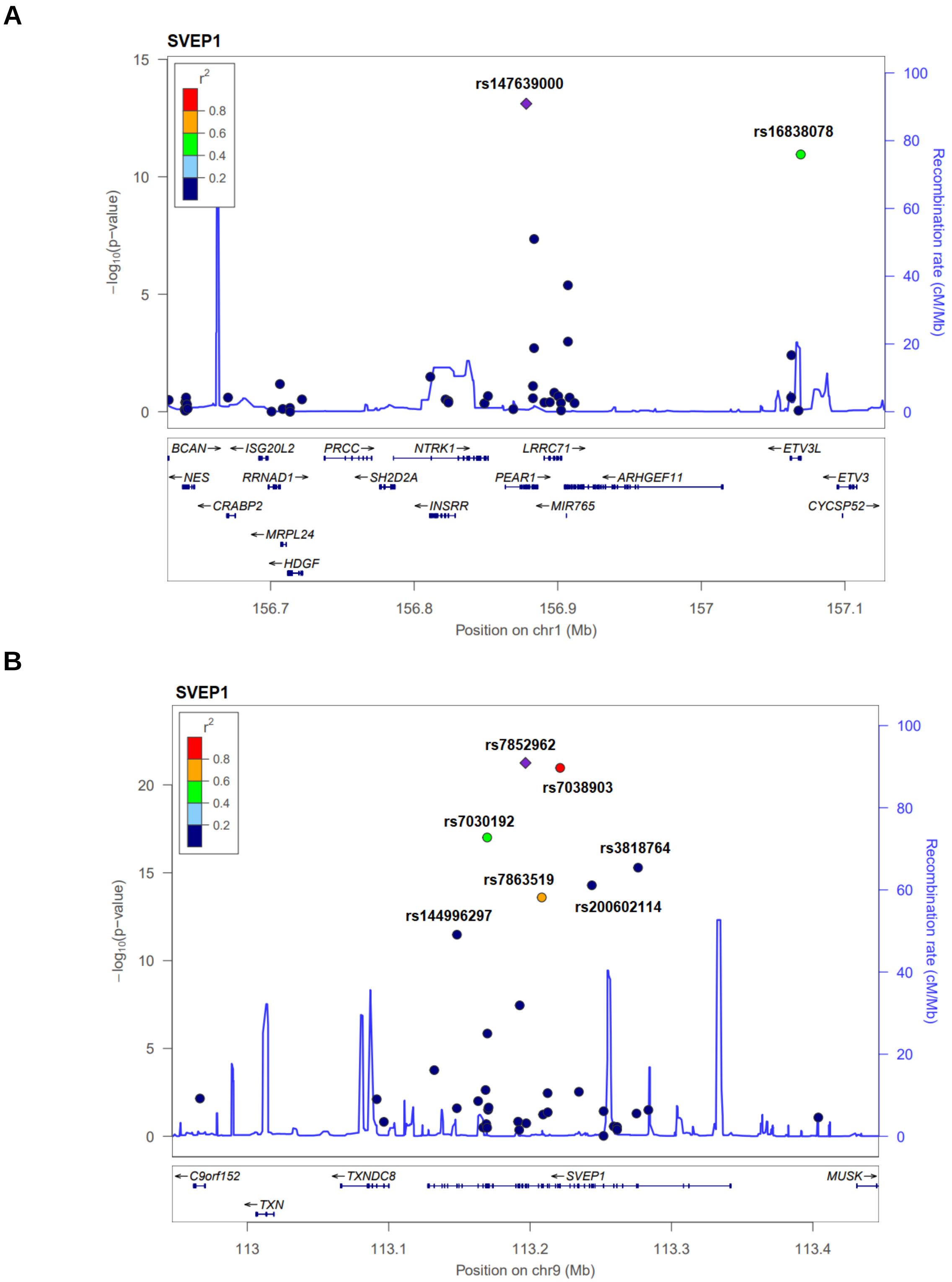

Supplementary Fig. 5. SVEP1 regional plots (LocusZoom) based on exome array variants at (**A**) chromosome 1 and (**B**) chromosome 9, using linkage disequilibrium (LD) data from the AGES-RS cohort. Each plot highlights study-wide significant pQTLs ($P < 1.92\times10^{-10}$, two-sided) pQTLs with chromosomal location (the megabase position is based on the human genome

coordinates version GRCh37/hg19) at the x-axis and -log(P-value) at the y-axis. Datapoints (SNPs) are colored based on their correlation (r^2) with the top SNP, which has the smallest P-value in the region. The fine-scale recombination rates are shown in light blue with genes highlighted below.

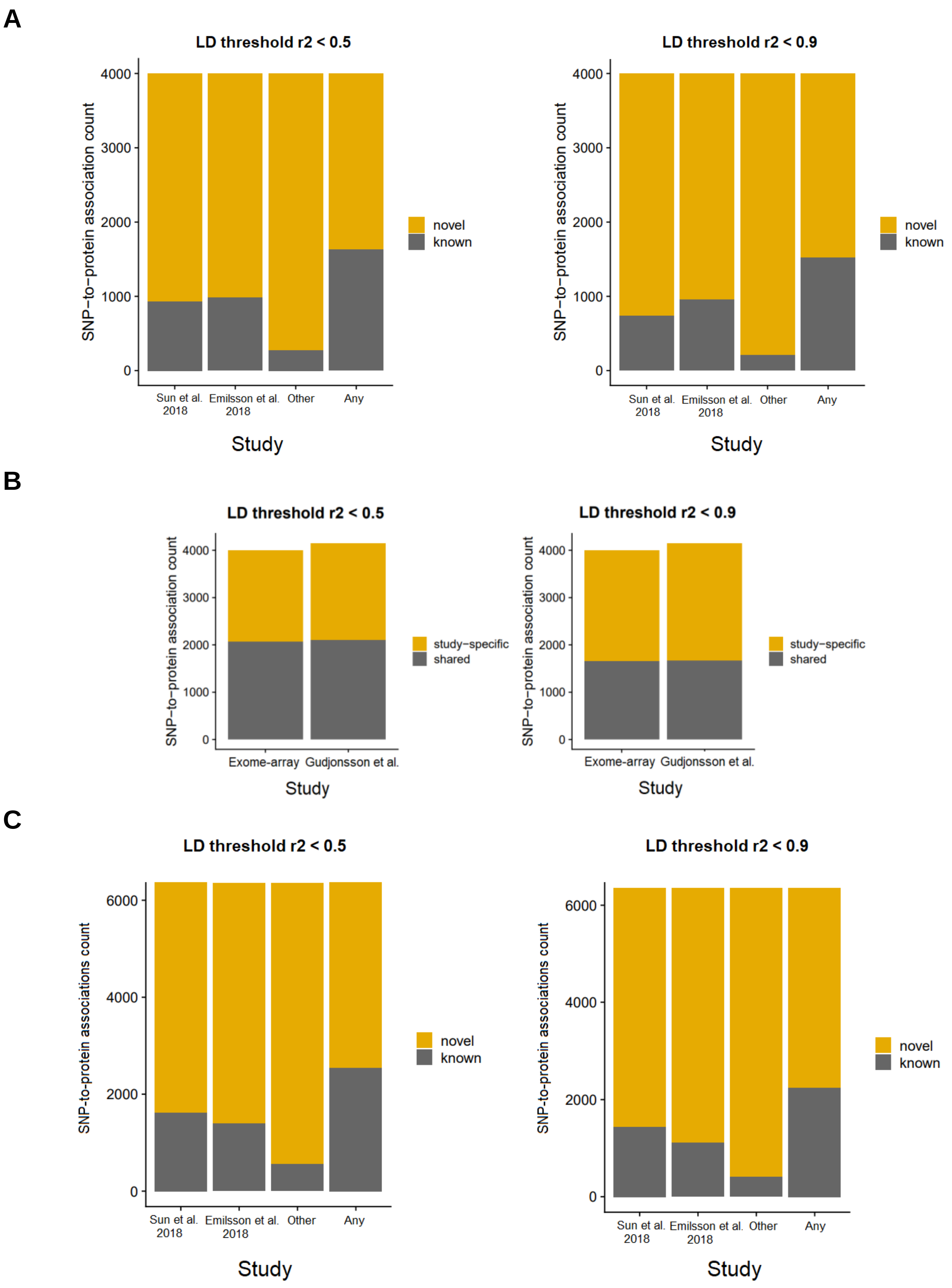

Supplementary Fig. 6. Assessing the current study's novelty in terms of pQTL discovery in comparison to publicly available findings. (**A**) The comparison of all conditionally independent SNP-to-protein associations in the exome-array paper to all 19 external studies found in the

public domain (listed in Supplementary Data 6) including for instance Sun et al. (2018)[2] and Emilsson et al (2018)[1]. The label -Other- refers to any proteogenomic study in the public domain that is not Sun et al. (2018)[2] or Emilsson et al (2018)[1]. The term -Any- refers to all 19 proteogenomic studies that have been published to date (Supplementary Data 6). Two different LD thresholds were used for this comparison: LD of r^2<0.5 (left panel) and r^2<0.9 (right panel). (**B**) All conditionally independent study-wide significant SNP-to-protein associations in the current exome-array were compared to those reported in our companion GWAS paper[3] at two different LD thresholds: LD of r^2<0.5 (left panel) and r^2<0.9 (right panel). (**C**) Each companion (GWAS and exome-array) study's combined unique and common independent study-wide significant pQTLs were compared to published proteogenomic studies for novelty at different LD thresholds: LD of r^2<0.5 (left panel) and r^2<0.9 (right panel). The label -Other- refers to any proteogenomic study in the public domain (Supplementary Data 6) that is not Sun et al. (2018)[2] or Emilsson et al (2018)[1]. The term -Any- refers to all 19 proteogenomic studies that have been published to date (Supplementary Data 6). The barplots in (**A**-**C**) indicates whether or not a matching pQTL association has previously been reported (known) or not (novel).

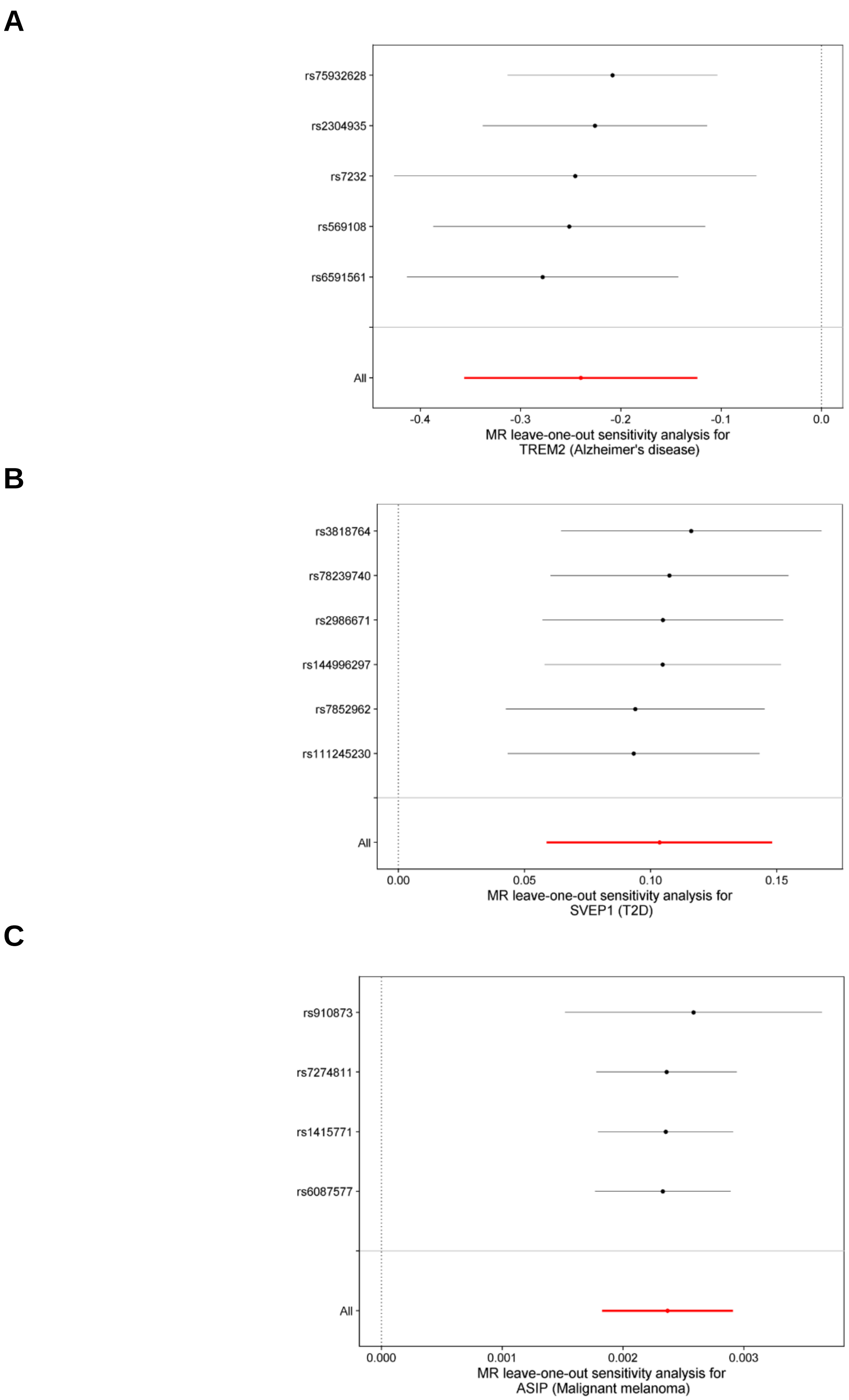

Supplementary Fig. 7. Leave-one-out plots for the MR analyes of (**A**) TREM2 (one *cis* plus four *trans* pQTLs), (**B**) SVEP1 (six *cis* pQTLs) and (**C**) ASIP (four *cis* pQTLs) to assess if the causal estimates are reliant on any single SNP instrument for a given MR test. The y-axis

denotes the individual pQTL instruments, while the x-axis denotes the the estimated effect as β-coefficient = log(odds ratio), along with 95% confidence intervals.

References

1 Emilsson, V. *et al.* Co-regulatory networks of human serum proteins link genetics to disease. *Science* **361**, 769-773, doi:10.1126/science.aaq1327 (2018).

2 Sun, B. B. *et al.* Genomic atlas of the human plasma proteome. *Nature* **558**, 73-79, doi:10.1038/s41586-018-0175-2 (2018).

3 Gudjonsson, A. *et al.* A genome-wide association study of serum proteins reveals shared loci with common diseases. *bioRxiv*, 2021.2007.2002.450858, doi:10.1101/2021.07.02.450858 (2021).
