# Supplementary material for: Coding and regulatory variants are associated with serum protein levels and disease
Source: Nat Commun. 2022 Jan 25;13:481. doi: 10.1038/s41467-022-28081-6 (PMC8789809; doi:10.1038/s41467-022-28081-6)
Supplement: Supplementary file 3 — Description of Additional Supplementary Files [file 41467_2022_28081_MOESM3_ESM.pdf]

Supplementary Data Legends

File Name: **Supplementary Data 1**
Description: Exome array GWAS results for protein quantitative trait loci (pQTLs) with minor allele frequencies (MAF) > 0.001 at P-value < $1x10^{-6}$ (two-sided) prior to any conditional analysis (see Supplementary Data 2). Allele 1 is the effect allele. All variant locations are according to the GrCh37 build of the human genome. The pQTL is cis acting if the SNP is within 300kb up- or downstream of the gene boundaries or within the gene. In contrast, trans pQTLs are outside the 300kb window.

File Name: **Supplementary Data 2**
Description: All independent genetic signals based on findings in Supplementary Data 1, performed using a stepwise conditional and joint multiple SNP association (linear regression) analysis using the GCTA-COJO (v1.92.4beta2) software for each protein analyte as described in Methods. The Beta coefficient (log(odds ratio)), Standard error and P-value columns show the pre-conditional regression coefficients (effect size, standard error and two-sided P-value), while the appended J signifies the coefficients in the conditional-joint analysis. The table presents all pQTLs for the conditional-joint analysis at P-value J < $1x10^{-6}$ (two-sided). All variant and gene locations are according to the GrCh37 build of the human genome. The gene containing the exome array variant is denoted by the column title Gene, while a variant is denoted by N/A if it is intergenic.

File Name: **Supplementary Data 3**
Description: We used the Ensemble Variant Effect Predictor (VEP) (https://www.ensembl.org/info/docs/tools/vep/index.html) to generate various pathogenicity prediction scores for all study-wide significant pQTLs in Supplementary Data 2 that included SIFT, PolyPhen, Likelihood Ratio Test (LRT), Variant Effect Scoring Tool (VEST), MutationAssessor and MutationTaster. GTEx gene expression data (https://www.gtexportal.org) related to potential tissue of origin of individual proteins (based on the aptamer). The Z>9.24 represents the top 0.5% of all tissue-specific Z-scores for the proteins measured. Numerical output of tissue specificity. Column Data; shows the numerical output of tissue specificity for the top 0.5% (Z>9.24) in tissue specific expression. We note that the structural variant can either reside within the protein coding gene of interest (based on the aptamer) or within another gene. The gene containing the exome array variant is denoted by the column title Gene, while a variant is denoted by N/A if it is intergenic.

File Name: **Supplementary Data 4**
Description: The overlap of study-wide significant pQTLs (P < $1.92x10^{-10}$, two-sided) identified in the present study with previously detected risk loci for complex disease and clinical traits reported through the numerous GWAS studies published todate and stored at the PhenoScanner database (http://www.phenoscanner.medschl.cam.ac.uk/). The comparison was made to genome-wide significant disease risk loci only (P < $5x10^{-8}$, two-sided) and no SNP proxies applied, except when the lead pSNP was not in the query then we used the best proxy.

File Name: **Supplementary Data 5**
Description: Two-sample Mendelian randomization (MR) analysis of TREM2, SVEP1, and ASIP for causal relationship to a disease of interest, using the inverse variance weighted estimate. The MR-Egger regression is also shown to assess horizontal pleiotropy, as are Cochran's Q-statistics to estimate heterogeneity. As shown, cis-only (pQTL) genetic (pQTL) instruments, cis + trans genetic (pQTL) instruments, and trans-only genetic (pQTL) instruments were used in the MR analysis. Because TREM2 only has a single cis acting genetic instrument, a Wald ratio estimate for that effect was also performed. The effect estimate is the beta as log(odds ratio). Also shown are the standard error (SE) and P-values (two-sided). For the different outcomes we used large-scale GWAS associations (linear regressions) for LOAD, T2D, malignant melanoma and systolic blood pressure as described in Methods.

File Name: **Supplementary Data 6**
Description: Overview of previously published studies used for estimating the novelty of pQTLs reported in the current study.

File Name: **Supplementary Data 7**
Description: The current exome array study's locus level novelty in comparison to the GWAS by Gudjonsson et al. (2021) and all pQTLs reported in Supplementary Data 6. The locus level novelty of the two companion papers combined is also shown in comparison to all pQTL studies published to date (Supplementary Data 6).
